# Weight Status Determines the Impact of a School-Based Nutrition Education Intervention on Lifestyle Behaviors in Children

**DOI:** 10.3390/children11091093

**Published:** 2024-09-06

**Authors:** María L. Miguel-Berges, Alicia Larruy-García, Pilar De Miguel-Etayo, Andrea Jimeno-Martinez, Antonio Torres, Luis A. Moreno

**Affiliations:** 1 Growth, Exercise, Nutrition and Development (GENUD) Research Group, University of Zaragoza, C/Domingo Miral s/n, 50009 Zaragoza, Spain; alarruy@unizar.es (A.L.-G.); pilardm@unizar.es (P.D.M.-E.); a.jimeno@unizar.es (A.J.-M.); lmoreno@unizar.es (L.A.M.); 2 Instituto Agroalimentario de Aragón (IA2), C/Miguel Servet 177, 50013 Zaragoza, Spain; 3 Instituto de Investigación Sanitaria de Aragón (IIS Aragón), Avda. San Juan Bosco 13, 50009 Zaragoza, Spain; 4 Centro de Investigación Biomédica en Red-Fisiopatología de la Obesidad y Nutrición (CIBEROBN), (CB15/00043), Institute of Health Carlos III (ISCIII), Av. Monforte de Lemos, 3-5, Pabellón 11, Planta 0, 28029 Madrid, Spain; 5 School of Health Sciences Blanquerna, University Ramon Llull, 08022 Barcelona, Spain; ttorreshernandez@gmail.com

**Keywords:** childhood obesity, lifestyles, school-based interventions

## Abstract

Background/Objectives: This study investigated the impact of the FLUYE school-based intervention on children’s lifestyle behaviors, Mediterranean diet adherence, and emotional well-being in Spain. The objective was to promote healthy habits through nutrition education, physical activity, and emotional support within the school environment, with a focus on addressing the needs of both normal-weight and overweight/obese children. Methods: A total of 552 children aged 3 to 12 years participated in the study, with data collected at baseline (T0) and follow-up (T1). The intervention was designed to integrate health education into the school curriculum, emphasizing the development of personal competencies in diet, physical activity, and emotional well-being. The analysis included changes in dietary habits, screen time, physical activity, and psychosocial outcomes, with comparisons made between the normal-weight and overweight/obese groups. Results: Significant improvements were observed in water consumption and reductions in sugar-sweetened beverage intake across both weight groups. However, an increase in screen time, particularly among normal-weight children, highlighted ongoing challenges in reducing sedentary behavior. Adherence to the Mediterranean diet improved more significantly in the overweight/obese group, suggesting the program’s effectiveness in promoting healthier eating patterns among at-risk children. Emotional well-being and self-esteem also saw significant enhancements, with children reporting increased feelings of pride and positivity post-intervention. Conclusions: The FLUYE program effectively improved various aspects of children’s lifestyle behaviors, particularly in dietary habits and emotional well-being. These findings underscore the importance of comprehensive, school-based interventions that address both physical and psychosocial aspects of health, especially for children at higher risk for obesity.

## 1. Introduction

Childhood obesity is a growing concern worldwide strongly associated with adult obesity, with this relationship strengthening as children age [1]. Obesity in youth is linked to higher risks of adult diseases, morbidity, and mortality, independent of adult obesity [2]. For that reason, preventing childhood obesity may effectively mitigate these health risks, and this requires effective interventions to promote healthy eating behaviors among children. Currently, school-aged children have unhealthy habits regarding beverages consumption [3], Mediterranean diet adherence [4,5], and physical activity [6]. Studies have shown a significant rise in the consumption of sugar-sweetened beverages among children [7]. This trend, along with other factors, is contributing to the decline in adherence to the Mediterranean diet, which is known for its health benefits [7]. Additionally, there has been a notable decrease in physical activity levels along with an increase in screen time [6], exacerbating the risk of developing obesity and related health issues.

Numerous studies highlight the pivotal role of school-based interventions, involving families, as a promising strategy for reducing childhood obesity [8]. A meta-analysis assessing the impact of family-based interventions on childhood obesity concluded that such approaches significantly enhance effectiveness in promoting healthier lifestyles and reducing obesity in children [9]. Schools are ideal for these initiatives due to compulsory education, substantial daily time spent there by children, frequent provision of meals, physical education, and structured environments facilitating intervention implementation. Additionally, schools enable a broad reach and sustainability through teaching staff involvement [10]. A survey in the United States found that parents most frequently perceived schools, more than healthcare professionals and the government, as having had a “great responsibility” for reducing childhood obesity. Up to 65% of parents believed that schools must play a significant role in efforts to curb obesity [11].

Despite numerous programs developed to prevent childhood obesity, schools in Spain do not adequately address healthy lifestyle education or physical activity within their curricula. While schools are ideal intervention sites due to their structured environments and broad reach, the Spanish educational system currently fails to cover essential aspects of promoting healthy behaviors and increasing physical activity among children. This gap highlights the need for a more comprehensive integration of health education into school programs [12,13]. Furthermore, there is a scarcity of studies in Spain evaluating the effect of school-based interventions on children’s health behaviors, underscoring the need for further research in this field.

Some research has shown that prevention programs targeting the general population tend to be more effective for children with overweight or obesity [14]. This study therefore aims to investigate changes in lifestyle behaviors, adherence to the Mediterranean diet, and well-being among children categorized by weight status after a school-based intervention in Spain.

## 2. Methods

### 2.1. Design and Participants

A school health promotion program named “Alimentando el cambio” was conducted on preschool and school-age children. The intervention materials aiming to promote healthy lifestyles among students attending schools were produced by the Trilema Foundation in Spain and are named FLUYE (https://www.proyectofluye.com/ (accessed on 21 March 2024)). The participation rate of schools in the program was 69.2%, with participating schools located in the cities of Valencia, Madrid, Toledo, Soria, Zamora, Sevilla, and Albacete, comprising a total of 10 schools. A total of 1075 children aged 3 to 12 years were included in the study, with a participation rate of 64.3% [15]. Baseline (T0) data were collected between January 2020 and March 2020 and follow-up data (T1) were collected in October 2021. Information on physical activity, screen time, sleep duration, beverage consumption, adherence to the Mediterranean diet, emotional well-being, self-esteem, and sociodemographic and socioeconomic characteristics of preschool and school-age children was obtained through parental self-reported questionnaires, completed by fathers, mothers, and grandparents, among others. For this analysis, only 552 participants (48.4% girls) were included as they met the requirement of having responded to the questionnaires at T0 and T1. Written informed consent for the participation of their children was obtained from parents or legal guardians. Ethical approval for the study was obtained from the Aragon Committee of Ethics in Clinical Research (CEICA).

### 2.2. Intervention Program

The FLUYE program is an innovative school-based nutrition education intervention, designed to integrate nutrition into the school curriculum in Spanish schools, through the development of personal competencies. The program aims to teach children, aged 3 to 12 years, to care for themselves, improve their health, and enhance their well-being. It focuses on fostering healthy habits related to diet, hydration, personal hygiene, and physical activity, both at school and at home. Additionally, FLUYE emphasizes emotional balance, self-esteem, respect, and values, aiming to create long-term health improvements and a happier, ethically committed future generation. Educational materials and support for teachers, students, and families were provided to reinforce these habits. The intervention was conducted by the teachers themselves, who were provided with educational materials and support to reinforce these habits for students and families.

### 2.3. Anthropometric Measures

Anthropometric assessments were conducted by trained researchers in accordance with the International Society for the Advancement of Kinanthropometry (ISAK) Protocol [16]. Body weight was ascertained with participants wearing only underwear and no shoes, utilizing an electronic scale (SECA 877, SECA, Hamburg, Germany), precise to 0.1 kg, while body height was measured with a telescopic height (SECA 213, Hamburg, Germany), accurate to 0.1 cm. Body mass index (BMI) was derived by dividing weight (in kilograms) by height (in meters) squared. BMI z-scores were computed following the method outlined by Cole et al. [17]. Additionally, waist and hip circumference were measured to the nearest millimeter using an inelastic tape measure (Cescorf, Porto Alegre, Brazil), with participants standing in an upright position.

### 2.4. Socioeconomic Variables

The questionnaire encompassed inquiries regarding parental educational attainment and professional qualifications. Both maternal and paternal education levels, quantified in terms of years of schooling, were categorized into four distinct tiers: no studies, basic studies, vocational education, and university education. Maternal education, acknowledged as a robust proxy indicator of familial socioeconomic status [18], was selected as the primary variable for analysis.

### 2.5. Beverages and Dairy Products Consumption

Assessment of beverages and dairy products consumption utilized a semi-quantitative frequency questionnaire, adapted from the validated food frequency questionnaire employed in the ToyBox study [19]. In this study, ten distinct beverages and dairy products categories were identified and analyzed: (1) water, (2) sugar-sweetened beverages, (3) light beverages, (4) pure juices, (5) packaged juices, (6) smoothies, (7) milk, (8) sweetened milk, (9) natural yogurt, and (10) sugar yogurt.

### 2.6. Movement Behaviors: Physical Activity, Screen Time, and Nighttime Sleep

Physical activity (PA) was evaluated using a questionnaire that assessed sports participation, measured as the number of hours per week children engaged in one or two sports activities. This method of assessing PA through “sports participation” has been previously identified in European studies as showing the strongest association with moderate to vigorous physical activity as measured by accelerometers [20]. 

Screen time, encompassing television and computer usage, was evaluated by inquiring about the total duration of screen time on both weekdays and weekends. Responses were recorded regarding minutes spent watching television (including videos and DVDs) and engaging in computer activities per day. Response options included “never”, “<30 min/day”, “30 min to 1 h/day”, “1–2 h/day”, “3–4 h/day”, “5–6 h/day”, “7–8 h/day”, “8 h/day”, and “more than 8 h/day”. These categorical variables were then converted into minutes per day for weekdays and weekends separately. Additionally, responses were aggregated into two categories: ≤1 h/day and >1 h/day for preschool children, and ≤2 h/day and >2 h/day for school-age children, in accordance with sedentary behavior recommendations outlined by the World Health Organization [21] and Canadian guidelines [22]. 

Nighttime sleep was reported by parents, indicating the average number of hours and minutes their child slept per night. This information was provided separately for weekdays and weekends.

### 2.7. Mediterranean Diet Adherence

Adherence to the Mediterranean diet was evaluated using an index, derived from a 16-question test from a questionnaire entitled KIDMED [9]. This questionnaire comprised 12 queries regarding the frequency of food consumption and 4 inquiries regarding food intake habits characteristic of the Spanish Mediterranean diet. In total, there were 16 items answered by parents with two options: yes or not. Questions having a negative connotation with respect to the Mediterranean diet were assigned a value of −1, and those with a positive aspect +1. The cumulative score ranged from 0 to 12. Subsequently, the scores were classified as follows: scores < 3 indicated a diet significantly deviating from the Mediterranean diet model, scores of 3–7 indicated an acceptable diet needing improvement, and scores > 8 indicated an adequate adherence to Mediterranean diet. For our analyses, as we required a dichotomous variable, the score was reclassified into two categories: low/medium adherence for a score less than 8 and high adherence for a score greater than or equal to 8.

### 2.8. Emotional Well-Being and Self-Esteem

Psychosocial well-being was assessed with questions on emotional wellbeing, self-esteem and family life, based on three subscales of the “KINDLR questionnaire”, a validated instrument for measuring health-related quality of life in children and adolescents [23]. The questionnaires including 12 questions were filled by the parents. Responses included were: “not at all”, “almost never”, “sometimes”, “often”, and “always”.

### 2.9. Statistical Analysis

Predictive Analytics Software version 20 (IBM SPSS Statistics for Windows) was utilized for data analysis. Statistical analyses were stratified by BMI categories. Initially, continuous variables were analyzed using *t*-tests, while categorical variables were assessed using the χ2 test. Analysis of covariance (ANCOVA) was conducted to explore differences between each total screen time category for the BMI. A significance threshold of *p* < 0.05 was applied to all statistical tests, and corresponding *p*-values were interpreted accordingly.

## 3. Results

The demographic and anthropometric characteristics of the study participants in both years of the FLUYE program are summarized in Table 1. A total of 552 children participated, with 51.6% being boys and 48.4% girls. Body mass index (BMI) categories indicate that 75.7% of the participants were classified as having a normal weight, while 24.3% had overweight or obesity.

Table 2 shows changes in beverage consumption between the normal-weight and overweight/obese groups at T0 and T1. Normal-weight children showed a significant increase in water intake, while the overweight/obese group did not. Both groups had significant decreases in sugar-sweetened beverage consumption. Light-beverage consumption increased significantly in the normal-weight group, but not in the overweight/obese group. Normal-weight children had a modest increase in natural juice consumption, while the overweight/obese group had a slight decrease. Packed juice consumption decreased significantly in the normal-weight group but increased in the overweight/obese group. Smoothie consumption increased significantly in both groups. Milk, sweetened milk, natural yogurt, and sugar yogurt consumption decreased significantly in both groups.

Table 3 presents the changes in physical activity, screen time, and sleep habits in children categorized as having normal weight and overweight/obesity before (T0) and after (T1) their participation in the FLUYE Program. No significant changes were observed in physical activity levels between the groups. Screen time increased significantly, with weekday television viewing rising in the normal-weight group and both groups experiencing significant increases in PC use on weekdays and weekends. Total screen time showed significant increases on weekdays for both groups. Sleep habits showed significant decreases in nighttime sleep duration on weekdays for both groups, with no significant changes noted in weekend sleep duration. These trends were more pronounced in the normal-weight group compared to the overweight/obese group.

Table 4 describe of changes in adherence to the Mediterranean diet pattern in children at T0 and T1 of participation in FLUYE. Concretely, a statistically significant difference was observed in adherence patterns between the normal-weight and overweight/obese groups. Among participants initially categorized as having low/medium adherence to the Mediterranean diet, 49.5% in the normal-weight group and 50.4% in the overweight/obese group maintained this level of adherence over time. Conversely, those transitioning from low/medium to adequate adherence were significantly higher in the overweight/obese group compared to the normal-weight group (16.7% vs. 19.4%, *p* = 0.004). While the transition from adequate to low/medium adherence was similar between the two groups, a notable difference emerged in transitioning from adequate to adequate adherence, with a higher proportion observed among normal-weight participants (22.9%) compared to those in the overweight/obese group (16.3%). Changes in adherence to the Mediterranean diet between normal-weight and overweight/obese groups shows no significant differences between the groups (*p* = 0.356).

Table 5 describes changes in children’s well-being and self-esteem before (T0) and after (T1) their participation in FLUYE. Significant differences were observed between the normal-weight and overweight/obese groups concerning emotional well-being and self-esteem post participation. Specifically, 91.8% of normal-weight children reported laughter and having fun after the program, compared to 89.4% of overweight/obese children. Most children showed improvements or remained stable in emotional well-being. Self-esteem increased significantly in both groups, with many children feeling proud and on top of the world after the program. 69% of normal-weight children reported having good ideas at both time points, a statistically significant result, while the overweight/obese group did not show a significant change. Both groups felt comfortable with their parents before and after the program, but normal-weight children reported higher comfort at home (95.9% vs. 92.3%, *p* = 0.016). Significant differences emerged in arguments at home and feelings of overprotection, with fewer normal-weight children reporting arguments and a higher percentage of overweight/obese children feeling overprotected (*p* = 0.000 and *p* = 0.018, respectively).

## 4. Discussion

This study demonstrates the positive impact of the FLUYE school-based program on children’s lifestyle behaviors, adherence to the Mediterranean diet, and emotional well-being in Spain. Significant improvements were observed in water consumption and reductions in sugar-sweetened beverage intake across both weight groups. Although physical activity levels remained unchanged, screen time increased notably, particularly among normal-weight children. Adherence to the Mediterranean diet showed better improvement in the overweight/obese group. The program also significantly enhanced emotional well-being and self-esteem, highlighting the importance of addressing both physical and psychosocial health in interventions.

### 4.1. Beverage and Dairy Consumption

Recent studies observed that school-aged children are increasingly consuming sugar-sweetened beverages [24]. This trend is particularly evident in the rising intake of sugar-sweetened beverages and decreasing adherence to healthier options such as water and milk (19). However, our findings indicate that the normal-weight group showed a significant increase in water intake and a significant decrease in the consumption of sugar-sweetened beverages (SSB) after the FLUYE program. When comparing the changes between the two groups, it is noteworthy that the increase in water intake was more pronounced in the overweight/obese group (0.25) compared to the normal-weight group (0.06), despite the lack of statistical significance in the overweight/obese group. On the other hand, both groups significantly reduced their consumption of sugar-sweetened beverages, but the magnitude of this reduction was comparable between the normal-weight (−0.05) and overweight/obese (−0.06) groups. This indicates that the program effectively reduced SSB consumption across different weight statuses, although the changes in water intake varied. This increase in water consumption aligns with studies emphasizing the benefits of water for hydration and reduced calorie intake [25]. Conversely, both groups showed a significant decrease in the consumption of sugar-sweetened beverages. We can be confident that adherence to these good habits in school-age children is encouraging given the relevant association between high SSB intake and obesity, type 2 diabetes, and cardiovascular diseases in adulthood [26].

On the contrary, we found a significant increase in light-beverage consumption among the normal-weight group from T0 to T1, whereas the overweight/obese group experienced a less pronounced increase that did not reach statistical significance. Specifically, the normal-weight group showed a mean difference of 0.02 compared to 0.01 in the overweight/obese group. We think that there is a trend in children and adolescents towards beverages perceived as healthier alternatives to SSBs. The general population and, in particular, many parents believe that light beverages are a healthier option for their children, which could explain the observed increase [27]. This misconception about the health benefits of light beverages can lead to higher intake among children, associating it with weight gain and metabolic issues, similar to those associated with SSBs [28].

Regarding dairy products, the consumption of smoothies increased significantly in both groups from T0 to T1. However, there was a significant decrease in the consumption of milk, sweetened milk, natural yogurt, and sugar yogurt in both groups. The normal-weight group had a mean decrease in milk consumption of −0.40 compared to −0.51 in the overweight/obese group. This reduction in traditional dairy intake might be partially driven by the rise of smoothie consumption, which parents might view as a healthier alternative because they incorporate fruits and vegetables, but these drinks should not replace traditional dairy products [29]. The FLUYE program’s positive impact on reducing the consumption of sugar-sweetened beverages and increasing water intake is commendable. Still, the unintended decline in dairy consumption suggests the need for a more balanced approach in future interventions to ensure comprehensive nutritional benefits. The study by Nury et al. [30] adds to the existing evidence on school based nutrition approaches to prevent obesity. However, it also reiterates that approaches focusing only on the school setting lead to marginal changes and are unlikely provide the solution to the public health problem of childhood obesity. To effect change, school-based interventions need to be accompanied by intervention approaches targeting all environments within which children are situated, and by wider changes to the food system and at a societal level.

### 4.2. Movement Behaviors

The results of movement behaviors revealed significant changes in screen time and sleep habits among children, but no notable changes in physical activity levels. Specifically, screen time significantly increased for both the normal-weight and overweight/obese groups on weekdays and weekends. For instance, weekday television viewing increased significantly more in the normal-weight group (Mean Dif: 8.83 ± 56.98) compared to the overweight/obese group (Mean Dif: 3.48 ± 53.72, *p* = 0.012 vs. *p* = 0.578). These findings align with recent trends observed in various studies, underscoring the complex interplay between physical activity, screen time, and sleep among children.

The lack of an effective impact children’s physical activity is consistent with previous research. Dobbins et al. [31], in a systematic review where they summarized the evidence of the effectiveness of school-based interventions in promoting physical activity, concluded that school-based interventions often face challenges in significantly altering physical activity behaviors due to various barriers, including environmental constraints and competing academic demands. Our results also reflect today’s reality, where children’s screen time has been steadily increasing, exacerbated by the growing availability of digital devices and online entertainment options [32]. Furthermore, the reduction in nighttime sleep duration on weekdays was significant for both groups. However, the decrease was slightly more pronounced in the overweight/obese group (Mean Dif: −0.35 ± 1.05) compared to the normal-weight group (Mean Dif: −0.22 ± 1.07), with *p*-values of 0.001 and 0.000, respectively. These results are consistent with findings linking screen time with sleep disturbances among children [33]. These results underscore the complex interplay between lifestyle behaviors and intervention programs, highlighting the need for comprehensive strategies to promote healthy habits among children.

### 4.3. Mediterranean Diet Adherence

The results indicated a transition from low/medium to adequate adherence significantly higher in the overweight/obese group compared to the normal-weight group. This shift indicates a positive impact of the FLUYE program in encouraging better dietary practices among children with overweight/obesity. However, the comparison of mean differences between the groups reveals no significant change between the normal-weight and overweight/obese groups in terms of maintaining or improving their adherence to the Mediterranean diet. Previous studies have shown that school-based interventions can effectively improve dietary habits and increase adherence to healthier eating patterns, such as the Mediterranean diet [34]. It is worth noting that the Mediterranean diet is renowned for its numerous health benefits, including reduced risk of chronic diseases and improved weight management [4,35].

### 4.4. Well-Being and Self-Esteem

Significant improvements were observed in both emotional well-being and self-esteem among participants, indicating the positive effects of the intervention.

The program led to a high percentage of children reporting increased laughter and enjoyment, with 91.8% of normal-weight children and 89.4% of overweight/obese children expressing these positive emotions post-intervention. In terms of emotional well-being, no significant differences were found between the normal-weight and overweight/obese groups (*p* = 0.509) for children who reported “laughing and having a lot of fun”. However, there was a significant increase in children feeling “on top of the world” in the normal-weight group compared to the overweight/obese group (*p* = 0.000).

These results are consistent with a meta-analysis about school-based interventions which suggested that can enhance children’s emotional well-being by providing supportive environments and engaging activities [36]. The slight difference between the normal-weight and overweight/obese groups highlights the importance of tailored approaches to ensure all children equally benefit from such programs. The improvement in emotional well-being is particularly important as it has implications for children’s development. Emotional well-being is closely linked to mental health, academic success, and social relationships [37]. Children who experience positive emotions are more likely to engage in school activities, form healthy relationships, and exhibit resilience in the face of challenges. Moreover, children with overweight and obesity often face additional emotional and social challenges [38]. Therefore, interventions such as FLUYE that promote emotional well-being can be particularly beneficial for these groups.

Findings on children’s self-esteem demonstrated a significant increase in feelings of pride, self-worth, and overall positivity post-program participation among both the normal-weight and overweight/obese groups. Notably, a significant proportion of children reported feeling “on top of the world” after the program, indicating boosted confidence and emotional well-being. This increase in self-esteem is crucial as higher self-esteem is associated with better academic performance, improved social relationships, and overall mental health [39]. Children with high self-esteem are more likely to engage actively in classroom activities and maintain positive social interactions [40].

Our results align with existing literature suggesting that interventions promoting healthy lifestyle behaviors can positively impact children’s self-esteem, fostering a sense of capability and resilience. Overall, the FLUYE program’s impact on emotional well-being and self-esteem underscores the importance of integrating emotional health components into school-based interventions to promote holistic development in children.

### 4.5. Strengths and Limitations

This study has several strengths. The longitudinal design allows for the observation of changes over time, providing a more accurate assessment of the FLUYE intervention’s effectiveness. The use of standardized anthropometric measurements, including validated protocols for measuring weight, height, and circumferences, increases the precision and reliability of the collected data. The innovative nature of the FLUYE program, integrating nutrition education and health promotion into the school curriculum, presents a practical and sustainable solution for improving children’s habits. However, there are also limitations. The self-reported questionnaires completed by parents and caregivers may introduce recall and social desirability biases. While parental questionnaires provide valuable insights, they may not fully capture the nuances of children’s behaviors and emotional well-being. The lack of a control group limits the ability to establish a definitive causal relationship between the intervention and observed changes. Although the longitudinal design is a strength, follow-up was only possible in the lifestyle variables assessed through questionnaires and not the anthropometric assessment.

## 5. Conclusions

These results underscore the importance of school-based interventions in improving healthy lifestyle habits among children. The FLUYE program demonstrated a multidimensional impact on various lifestyle behaviors, including beverage consumption, physical activity, adherence to the Mediterranean diet, emotional well-being, and self-esteem. The positive changes observed highlight the necessity for comprehensive and tailored strategies to effectively promote healthy habits among all children, particularly those with overweight and obesity. Implementing such interventions within the school curriculum can foster a supportive environment that encourages the development of lifelong healthy behaviors, contributing to better overall health outcomes.

## Figures and Tables

**Table 1:** Total children participating in baseline and follow-up examinations in the FLUYE program (n = 552).

		**N**	**%**
Boys		285	51.6
Girls		267	48.4
Mother’s education			
	Required studies	85	15.4
	High School y/o FP	368	66.7
	University	97	17.6
	Without studies	2	0.4
	Overweight/obese	134	24.3
		Mean	SD
Age (years)		7.72	2.60
Body mass index (kg/m^2^)		17.58	3.09
Body mass index (z-score) *		0.49	1.19
Waist circumference (cm)		58.94	8.48
Hip circumference (cm)		68.89	11.27
Waist/height index		0.47	0.04

* According to the criteria established by Cole et al. [17]

**Table 2:** Changes in beverage consumption in children with normal weight or overweight/obesity from baseline to follow-up.

	Time 0	Time 1			Time 0	Time 1			
Portions per Day	Mean (SD)	Mean (SD)	Mean Dif	*p*-Value	Mean (SD)	Mean (SD)	Mean Dif	*p*-Value	*p*-Value *
Water	2.91 ± 1.08	2.97 ± 1.18	0.06 ± 1.13	**0.00**	3.13 ±1.11	3.38 ±1.14	0.25 ± 1.13	0.232	0.236
Sugar-sweetened beverages	0.14 ± 0.29	0.09 ± 0.18	−0.05 ± 0.24	**0.00**	0.15 ± 0.28	0.09 ± 0.15	−0.06 ± 0.22	**0.021**	0.759
Light beverages	0.03 ± 0.12	0.05 ± 0.15	0.02 ± 0.14	**0.00**	0.03 ± 0.010	0.04 ± 0.12	0.01 ± 0.09	0.447	0.549
Natural juices	0.30 ± 0.47	0.32 ± 0.44	0.02 ± 0.46	**0.00**	0.29 ± 0.53	0.27 ± 0.40	−0.02 ± 0.47	**0.00**	0.544
Packed Juices	0.31 ± 0.54	0.29 ± 0.39	−0.02 ± 0.47	**0.00**	0.26 ± 0.46	0.28 ± 0.41	0.02 ± 0.44	**0.00**	0.535
Smoothies	0.18 ± 0.48	0.52 ± 0.87	0.34 ± 0.70	**0.00**	0.22 ± 0.49	0.43 ± 0.91	0.21 ± 0.73	**0.00**	0.200
Milk	1.56 ± 0.86	1.16 ± 0.99	−0.40 ± 0.93	**0.00**	1.72 ± 1.03	1.21 ± 0.96	−0.51 ± 1.00	**0.00**	0.422
Sweetened milk	0.53 ± 0.81	0.40 ± 0.62	−0.13 ± 0.72	**0.02**	0.40 ± 0.74	0.32 ± 0.47	−0.08 ± 0.62	**0.00**	0.599
Natural yogurt	0.27 ± 0.38	0.25 ± 0.34	−0.02 ± 0.36	**0.00**	0.27 ± 0.37	0.22 ± 0.30	−0.05 ± 0.34	**0.00**	0.545
Sugar yogurt	0.45 ± 0.42	0.29 ± 0.36	−0.16 ± 0.39	**0.00**	0.50 ± 0.44	0.29 ± 0.37	−0.21 ± 0.41	**0.00**	0.378

Significant differences (*p* < 0.05) are shown in bold font. * The *p*-values in the “Mean Dif Comparison” column were calculated using an independent-samples *t*-test to compare the mean differences between the normal-weight and overweight/obese groups. The level of significance was set at *p* ≤ 0.05.

**Table 3:** Changes in movement behaviors in children with normal weight or overweight/obesity from baseline to follow-up.

	Normal Weight						Overweight/Obesity						
	Time 0		Time 1				Time 0		Time 1				Mean Dif Comparison (Normal Weight vs. Overweight/Obesity)
	Mean	SD	Mean	SD	Mean Dif	*p*-Value	Mean	SD	Mean	SD	Mean Dif	*p*-Value	*p*-Value
Physical Activity (min/day)	24.24	13.56	25.74	19.00	1.50 ± 6.98	0.391	27.22	17.29	25.30	17.51	−1.92 ± 12.79	0.577	0.211
Television (min/day)													
Weekdays	52.13	55.68	60.96	58.18	8.83 ± 56.98	0.012	60.37	57.43	63.85	49.75	3.48 ± 53.72	0.578	0.495
Weekend	123.06	100.93	129.66	88.15	6.60 ± 94.72	0.251	126.03	112.70	123.88	87.51	−2.15 ± 100.90	0.856	0.528
PC (min/day)													
Weekdays	24.36	47.46	45.94	54.31	21.58 ± 51.00	0.000	38.14	63.54	54.08	56.27	15.94 ± 60.01	0.012	0.474
Weekend	75.92	89.80	103.70	92.63	27.78 ± 91.23	0.000	102.75	94.51	124.46	102.37	21.71 ± 98.52	0.020	0.651
Total screen time (min/day)													
Weekdays	76.63	81.41	106.70	89.94	30.07 ± 85.79	0.000	99.31	91.18	117.04	75.31	17.73 ± 83.61	0.033	0.304
Weekend	199.91	149.89	230.91	142.17	31.00 ± 146.07	0.000	228.51-	158.09	245.36	148.72	16.85 ± 153.55	0.312	0.505
Nighttime sleep (hours/night)													
Weekdays	8.90	1.08	8.68	1.06	−0.22 ± 1.07	0.000	8.63	0.97	8.28	1.13	−0.35 ± 1.05	0.001	0.386
Weekend	9.53	1.18	9.46	1.13	−0.07 ± 1.16	0.333	9.35	1.24	9.02	1.32	−0.33 ± 1.28	0.013	0.134

Data are mean and standard deviation (quantitative variables). The *p*-values in the “Mean Dif Comparison” column were calculated using an independent-samples *t*-test to compare the mean differences between the normal-weight and overweight/obese groups The level of statistical significance is set at *p* ≤ 0.05.

**Table 4:** Description of changes in the adherence to Mediterranean diet in children at baseline and follow-up.

	Normal Weight			Overweight/Obesity			
Adherence to DMed (Total Score)	n	%	*p*	n	%	*p*	Changes between Groups (*p*) *
Low/Medium-Low/Medium	201	49.5	0.000	65	50.4	0.004	0.356
Low/Medium-Adequate	68	16.7		25	19.4		
Adequate-Low/Medium	44	10.8		18	14.0		
Adequate-Adequate	93	22.9		21	16.3		

* Statistical significance for differences between groups was determined using the chi-square test. The level of significance was set at *p* ≤ 0.05.

**Table 5:** Description of changes in children’s well-being and self-esteem in children from baseline to follow-up.

	Change of Habit (T1-T0)							
		Normal Weight			Overweight/Obesity			
		N	%	*p*-Value	N	%	*p*-Value	Changes between Groups (*p*) *
Emotional well-being.								
… He was laughing and had a lot of fun.	Y-Y	325	91.8	0.000	101	89.4	0.050	0.509
	N-Y	11	3.1		7	6.2		
	Y-N	12	3.4		3	2.7		
	N-N	6	1.7		2	1.8		
… He didn’t feel right to do anything.	Y-Y	0	0	0.925	0	0	0.978	0.925
	N-Y	5	1.5		2	2.2		
	Y-N	5	1.5		1	1.1		
	N-N	3/5	96.9		86	96.6		
… felt lonely.	Y-Y	0	0	0.997	0	0	0.999	0.997
	N-Y	1	0.3		1	1.1		
	Y-N	1	0.3		0	0		
	N-N	326	99.4		88	98.9		
… He felt insecure and anxious.	Y-Y	0	0	0.955	0	0	0.978	0.955
	N-Y	3	0.9		3	3.4		
	Y-N	5	1.5		0	0		
	N-N	318	97.5		86	96.6		
Self-esteem.								
… He was proud of himself.	Y-Y	222	65.3	0.001	67	65.7	0.002	0.665
	N-Y	38	11.2		10	9.8		
	Y-N	55	16.2		14	13.7		
	N-N	25	7.4		11	10.8		
… felt on top of the world	Y-Y	54	16.5	0.000	17	18.7	0.004	0.000
	N-Y	33	10.1		12	13.2		
	Y-N	72	22		17	18.7		
	N-N	168	51.4		45	49.5		
… He felt good about himself/herself.	Y-Y	273	79.1	0.001	71	70.3	0.005	0.001
	N-Y	20	5.8		15	14.9		
	Y-N	40	11.6		7	6.9		
	N-N	12	3.5		8	7.9		
… He had good ideas.	Y-Y	231	69	0.002	58	61.1	0.345	0.632
	N-Y	22	6.6		15	15.8		
	Y-N	64	19.1		16	16.8		
	N-N	18	5.4		6	6.3		
Family								
… felt comfortable with us as parents.	Y-Y	319	91.4	0.510	96	93.2	0.140	0.340
	N-Y	13	3.7		2	1.9		
	Y-N	16	4.6		4	3.9		
	N-N	1	0.3		1	1		
… At ease at home	Y-Y	325	95.9	0.155	96	92.3	0.016	0.213
	N-Y	6	1.8		2	1.9		
	Y-N	7	2.1		4	3.8		
	N-N	1	0.3		2	1.9		
… We argued at home.	Y-Y	7	2.1	0.000	1	1.1	0.376	0.001
	N-Y	10	3		5	5.3		
	Y-N	16	4.8		6	6.3		
	N-N	303	90.2		83	87.4		
… He felt overprotected by us.	Y-Y	43	12.9	0.000	31	33	0.018	0.001
	N-Y	35	10.5		15	16		
	Y-N	49	14.7		63	67		
	N-N	206	61.9		46	48.9		

* Statistical significance for differences between groups was determined using the chi-square test. The level of significance was set at *p* ≤ 0.05. Y-Y: answered yes at T0 and T1; N-Y: answered no at T0 and yes at T1; Y-N: answered yes at T0 and not at T1; N-N: answered no at T0 and T1.

## Data Availability

The data supporting the findings of this study are currently not available due to ongoing analyses and follow-up studies. However, data may be made available upon completion of these studies, subject to appropriate ethical considerations and institutional policies.
